# Geographical Distribution Patterns of Iodine in Drinking-Water and Its Associations with Geological Factors in Shandong Province, China

**DOI:** 10.3390/ijerph110505431

**Published:** 2014-05-19

**Authors:** Jie Gao, Zhijie Zhang, Yi Hu, Jianchao Bian, Wen Jiang, Xiaoming Wang, Liqian Sun, Qingwu Jiang

**Affiliations:** 1 Department of Epidemiology, School of Public Health, Fudan University, Shanghai 200032, China; E-Mails: agao1224@163.com (J.G.); stevenhyy@163.com (Y.H.); 13211020063@fudan.edu.cn (L.S.); 2 Key Laboratory of Public Health Safety, Ministry of Education, Shanghai 200032, China; 3 Laboratory for Spatial Analysis and Modeling, School of Public Health, Fudan University, Shanghai 200032, China; 4 Shandong Institute of Prevention and Control for Endemic Disease, Jinan 250014, China; E-Mails: shelloloh@126.com (W.J.); booew@163.com (X.W.)

**Keywords:** iodine, risk factors, spatial epidemiology, geographic information system

## Abstract

County-based spatial distribution characteristics and the related geological factors for iodine in drinking-water were studied in Shandong Province (China). Spatial autocorrelation analysis and spatial scan statistic were applied to analyze the spatial characteristics. Generalized linear models (GLMs) and geographically weighted regression (GWR) studies were conducted to explore the relationship between water iodine level and its related geological factors. The spatial distribution of iodine in drinking-water was significantly heterogeneous in Shandong Province (Moran’s *I* = 0.52, *Z* = 7.4, *p* < 0.001). Two clusters for high iodine in drinking-water were identified in the south-western and north-western parts of Shandong Province by the purely spatial scan statistic approach. Both GLMs and GWR indicated a significantly global association between iodine in drinking-water and geological factors. Furthermore, GWR showed obviously spatial variability across the study region. Soil type and distance to Yellow River were statistically significant at most areas of Shandong Province, confirming the hypothesis that the Yellow River causes iodine deposits in Shandong Province. Our results suggested that the more effective regional monitoring plan and water improvement strategies should be strengthened targeting at the cluster areas based on the characteristics of geological factors and the spatial variability of local relationships between iodine in drinking-water and geological factors.

## 1. Introduction

Iodine is the most important pathogenic factors responsible for endemic thyroid disease. Both insufficient and excessive iodine intake can cause thyroid hormone disorders. With the rapid progress in alleviating iodine deficiency, the iodine intake in developing countries has increased greatly [1,2]. The issue of excess iodine intake, however, continues to raise many concerns because of its various adverse effects [3,4]. For example, excessive iodine intake may cause thyroid goiter, overt hyper- and hypothyroidism, subclinical hyper- and hypothyroidism, autoimmune thyroid disease, loss of intelligence, *etc.* [5,6].

The naturally sources of iodine are found in food and water. China was the first country to report cases excess goiter caused by water-sourced iodine [7]. A national investigation showed that drinking-water with high iodine levels could be found in 11 provinces, mainly located in the middle of China, including Shandong, Hebei, Henan, Jiangsu, Anhui, and Shanxi provinces [8]. Drinking-water with high iodine levels was found in 288 townships of 19 counties including seven cities in Shandong Province [9]. The surveillance results conducted by the Shandong Institute of Prevention and Control for Endemic Disease indicated that there may be a strong spatial heterogeneity in the distribution of iodine in drinking-water in Shandong Province [9,10,11]. As drinking-water was identified as one of the main sources of iodine intake [12], understanding the spatial distribution and pattern of iodine in drinking-water may help the various health departments implement effective strategies of regional prevention to control the adverse effects of excess iodine intake.

In recent years, geographical information system (GIS) and spatial analysis techniques have been frequently used to describe the patterns of endemic diseases and pathogenic factors. The spatial analyses, such as spatial autocorrelation and cluster analysis are commonly used to characterize spatial epidemiology of endemic diseases [13,14,15,16]. Previous studies suggested that the iodine in drinking-water might have variability in different regions [17,18,19]. However, to the best of our knowledge, few studies have tried to explore the spatial pattern of iodine in drinking-water in Shandong Province, China [20].

Besides, the iodine concentration in drinking-water is mainly determined by the geological background, making it essential to investigate the determinants for high iodine, especially the geological factors. However, geological factors usually have spatial autocorrelation and show obvious spatial heterogeneity [21,22,23], so geographically weighted regression (GWR) was used to analyze the local spatial heterogeneity in the estimated relationships between iodine in drinking-water and geological factors, with the purpose of exploring possible clues to the geographical distribution of high levels of iodine in drinking-water.

## 2. Experimental Section

### 2.1. Study Area

**Figure 1:**
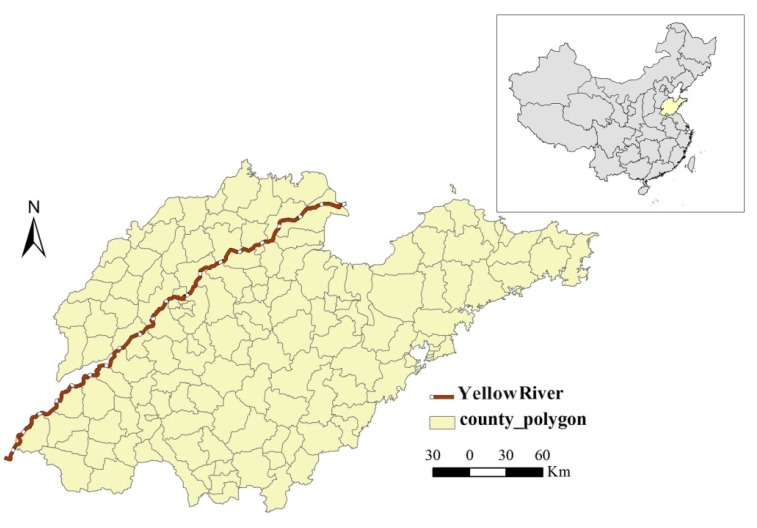
The location of study area—Shandong Province in China.

The study area, Shandong Province, located at latitude 34°22.9′ N–38°24.01′ N, longitude 114°47.5′ E–122°42.3′ E, is a coastal province in Eastern China with a population of approximately 98 million (Figure 1). It includes 140 counties with a total land area of 157,100 square kilometers. Shandong Province has a warm temperate monsoon climate, with the mean annual temperature of 11–14 °C. Geographically, Shandong Province can be divided three parts: the plain area from southwest to northwest, the mountainous and hilly area in the central, and the hilly area in the eastern part.

### 2.2. Data Collection

Data of iodine in drinking-water in Shandong Province were obtained from the Shandong Institute of Prevention and Control for Endemic Disease. The water samples were collected at the township scale. Each town was divided into five districts (*i.e.*, east, west, south, north and center). From each district, one village was randomly selected. For the villages with less than five wells, water samples from all available wells were collected. For those with more than five wells, the village was divided into five areas (*i.e.*, east, west, south, north and center) and one well in each area was selected. In the village where water was provided through pipelines, a sample was collected from the source of the water supply. In total, 108,164 water samples were collected from the 140 counties. The median iodine concentrations were calculated to represent the iodine concentration of the county. Water iodine was determined using the As-Ce catalytic spectrophotometric method for high water iodine [8]. Considering the high level of iodine in the water samples, we adopted the China Iodine Deficiency Disorder Laboratory standard for iodine measurement. Four geological factors, including hydrogeology, soil, topography, were selected in the present study based on the hypothesis that the Yellow River caused iodine deposits through flooding. The main type of hydrogeology, soil or topography in each county was defined as the type of the county. The geological factors were obtained from the Shandong Institute of Geological Survey, and the distance from each county to the Yellow River was calculated using the ArcGIS10 software (ESRI Inc., Redlands, CA, USA). Further information is provided in Table 1.

**Table 1:** Value assignment of the geological variables.

Geological Factors	Variables	Assignment
Hydrogeology	Unconsolidated rock water	1
	Fracturepore water in clastic rocks	2
	Fracturekarst water in Carbonate rocks	3
	basement rock fracture water	4
Soil	Brunisolic soil	1
	Cinnamon soil	2
	Moisture soil	3
	Shajiang Black soil	4
	Paddy soil	5
Topography	Plain	1
	Hills	2
DtY	Distance to Yellow River(Km)	--

### 2.3. Spatial Autocorrelation Analysis

Spatial autocorrelation analyses were performed using ArcGIS10 [24,25]. Global Moran’s *I* [26] was used to describe spatial autocorrelation and analyze the spatial distribution pattern of iodine in Shandong Province. Local Moran’s *I* [27] was applied to examine the local level of spatial autocorrelation and determine locations of clusters or hotspots. The significance of Moran’s *I* was assessed by employing Monte Carlo randomization. A statistically significant (*Z* ≥ 1.96) estimate of *I* indicates that neighboring counties have similar iodine concentration and are likely to cluster at county level.

### 2.4. Spatial Scan Statistic

The spatial scan statistic was performed using SaTScan V9.1.1 software (Kulldorff and Information Management Services, Inc., Boston, MA, USA) [27]. The Kulldorff’s statistic uses a moving window of pre-specified shape to identify high-risk or low-risk areas with statistically significant relatively high risk [28]. The null hypothesis assumed that all observations come from the same distribution. Under the alternative hypothesis, there is one cluster location where the observations have either a larger or smaller mean than outside that cluster. In present study, a normal distribution based model was used, because the iodine is continuous data. The geographic size of the window was limited to half of all observations [29]. The significance test of the identified clusters was based on comparing the likelihood ratio test statistics against a null distribution obtained from Monte Carlo Simulation. The number of permutations was set to 999 and the significance level was set at 0.05.

### 2.5. Detection of Spatial Relationships between Iodine Concentration and Geological Factors

To detect the spatial dependence relationship between iodine in drinking water and geological factors, the generalized linear models (GLMs) and geographically weighted regression (GWR) were used to build the global and local regression model between iodine concentration and geological factors(including hydrogeology, soil, topography and distance to Yellow River). GWR is a local version of spatial regression that generates parameters disaggregated by the spatial units of the analysis [30,31]. It allows assessment of the spatial heterogeneity in the estimated relationships between independent variables and the dependent variable. Unlike conventional GLMs, which may only produce a single regression equation to summarize global relationships between iodine in drinking water and geological factors, GWR can generate the spatial relationships that dynamically express the local spatial variation between them, because the regression coefficients of GWR are allowed to vary spatially. The SAS 9.3 software (SAS Institute Inc., Cary, NC, USA) was used for GLMs to set up the global dependence model and ArcGIS10 was used for GWR to set up the local regression model between iodine concentration and geographical factors.

## 3. Results

### 3.1. Iodine Concentration in Grounding-Water

According to the national standard “Determination and Classification of the Areas of High Water Iodine and the Endemic Areas of Iodine Excess Goiter” in China [32], there are 90 counties with a median water iodine of <10 μg/L, 31 counties with a median water iodine of 10–150 μg/L and 19 counties with a median water iodine >150 μg/L. 

**Figure 2:**
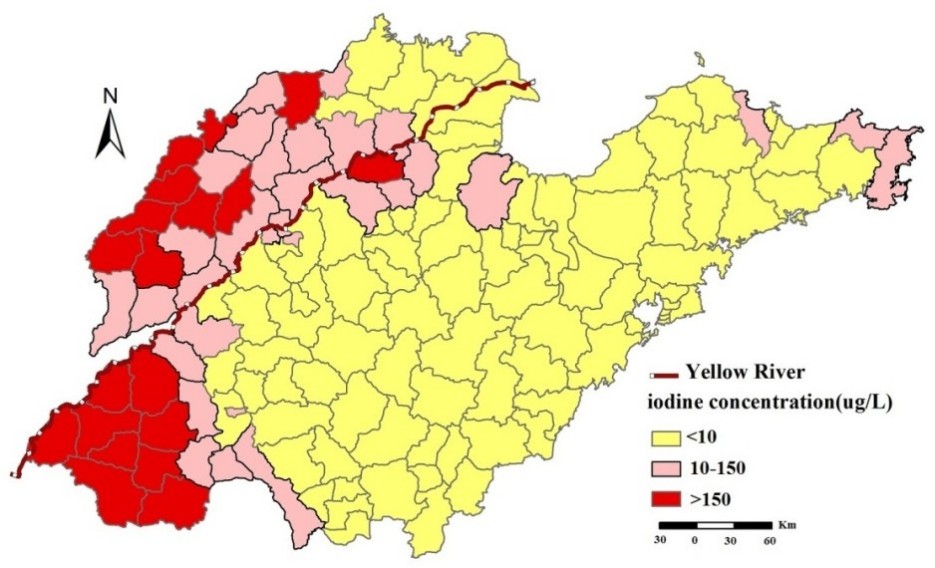
Distribution of iodine in drinking-water in Shandong province, China.

The excess iodine (>150 μg/L) regions were mainly located in southwest and northwest of Shandong Province. The “suitable” regions (10~150 μg/L) were mainly located in the counties along the Yellow River and the east of Shandong Province, while most counties were identified as iodine deficient (<10 μg/L) (Figure 2).

### 3.2. Spatial Autocorrelation Analysis

**Figure 3:**
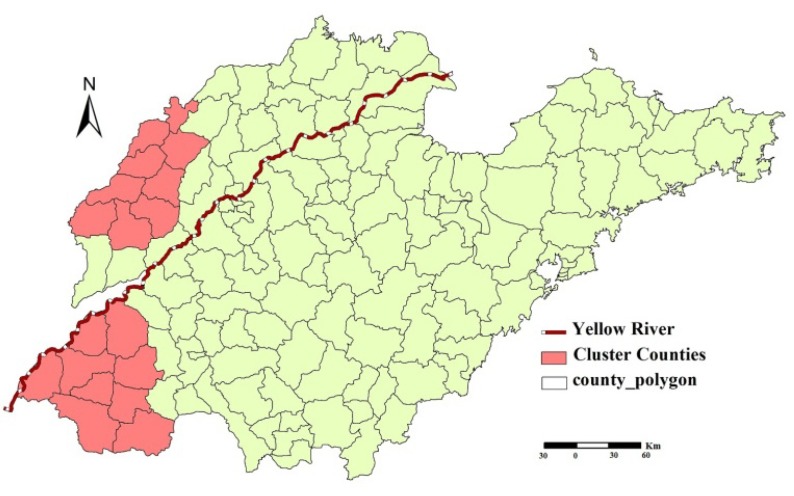
Spatial clusters detected by Local Moran’s *I* in Shandong Province, China.

Spatial distribution of iodine in drinking-water was spatially autocorrelated at the county level in Shandong Province, China (Moran’s *I* = 0.52, *p* < 0.001). Two significantly spatial clusters (hotspots) were identified using the Local Moran’s *I* (Figure 3). The hotspots included the 18 counties of Decheng, Wucheng, Pingyuan, Xiajin, Dongchangfu, Gaotang, Linqing, Chiping, Guanxian, Yuncheng, Quancheng, Dongming, Mudan, Juye, Dingtao, Chengwu, Caoxian and Shanxian.

### 3.3. Spatial Clusters of Iodine in Drinking Water

**Figure 4:**
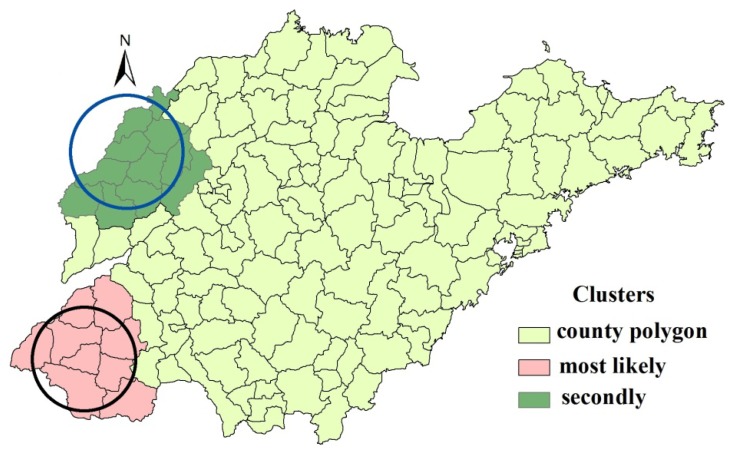
Spatial distributions of the detected clusters of high iodine areas in Shandong Province, China.

Figure 4 shows the statistically significant spatial clusters (including the most likely cluster and secondary cluster) for a high concentration of iodine in drinking-water identified by the purely spatial scan statistic. It can be seen that the most likely clusters including nine counties (southwest), and the secondary cluster included 11 counties (northwest). Table 2 summarizes the cluster information.

### 3.4. Global Spatial Dependence between Iodine Concentration in Drinking-Water and Geological Factors

Iodine concentration was significantly associated with geographical factors (*F* = 16.29, *p* < 0.001), and GLMs explained about 53.00% of the total variance of iodine concentration (*R*^2^ = 0.53) (Table 3). However, the test for the independence of residuals showed that it is not independent (Durbin-Watson *D* = 0.67) and the residual had an obvious spatial autocorrelation (Moran’s *I* = 0.64, *p* < 0.01), suggesting that the GLM does not fit the data well.

**Table 2:** Two clusters detected by spatial scan statistic.

Cluster center	Radius (km)	No. of counties in cluster areas	Log-likelihood ratio	*p* value
Dingtao	57.47	9	36.55	0.001
Xiajin	65.58	11	21.46	0.002

**Table 3:** Parameter estimates of GLMs.

Variable	DF	Parameter estimate	Standard Error	*T* Value	Pr > |*t*|
Intercept	--	0.3741	0.1153	3.2400	0.0015
Hydrogeology	Unconsolidated rock water	−0.0385	0.0533	−0.7200	0.4716
	Fracturepore water in clastic rocks	0.0245	0.0552	0.4500	0.6570
	Fracturekarst water in Carbonate rocks	0.0007	0.0541	0.0100	0.9900
	basement rock fracture water	--	--	--	--
Soil	Brunisolic soil	0.1264	0.1133	1.1200	0.2668
	Cinnamon soil	0.2398	0.1064	2.2500	0.0259
	Moisture soil	−0.0291	0.1028	−0.2800	0.7780
	Shajiang Black soil	0.1315	0.1451	0.9100	0.3662
	Paddy soil	--	--	--	--
Topography	Plain	−0.0359	0.0394	−0.9100	0.3629
	Hills	--	--	--	--
DtY	--	0.0005	0.0001	1.34	0.1833

Note: *R*^2^ = 0.53.

### 3.5. Local Spatial Dependence between Iodine Concentration and Geological Factors

Table 4 summarizes the results of GWR between iodine concentration and geographical factors, which indicated that there was large spatial variability in the parameter estimates across different regions. The *R*^2^ increased from 0.53 in GLMs to 0.63 in GWR, indicating that the GWR fitted the data better.

**Table 4:** The parameter estimates of the GWR model.

Variable	Minimum	1st Quartile	Median	3rd Quartile	Maximum
Intercept	0.2759	0.4264	0.5166	0.5469	0.7620
Hydrogeology	−0.0103	0.0071	0.0161	0.0233	0.0318
Soil	−0.1783	−0.1024	−0.0638	−0.0093	0.0069
Topography	0.0067	0.0355	0.0752	0.1038	0.1353
DtY	−0.0009	−0.0004	0.0005	0.0016	0.0022

Note: *R*^2^ = 0.63, *R*^2^ (adjusted) = 0.59.

**Figure 5:**
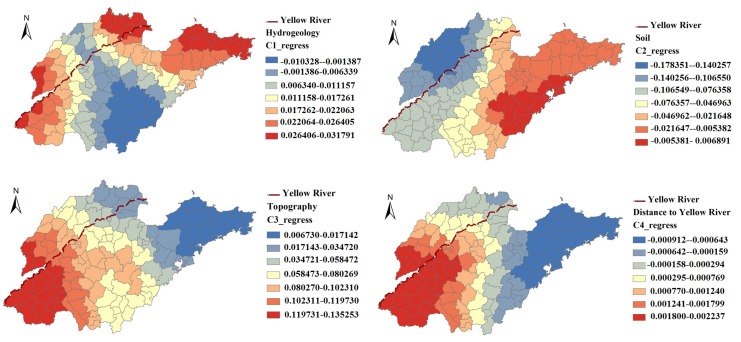
The coefficients of risk factors in Geographically Weighted Regression.

**Figure 6:**
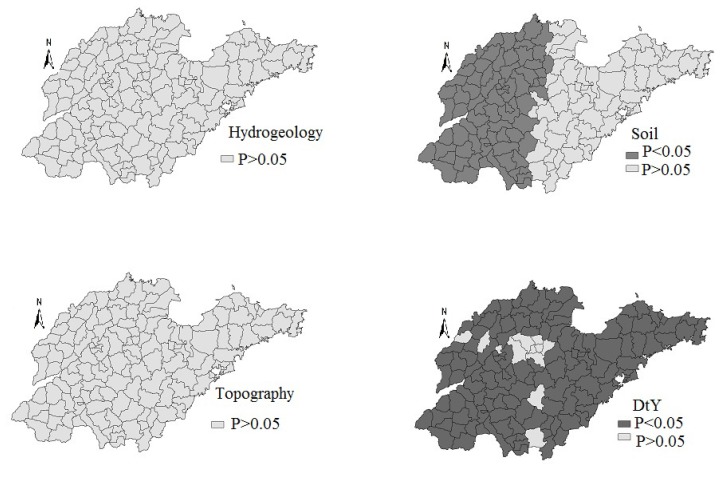
The *p* values of risk factors in geographically weighted regression.

Figure 5 and Figure 6 showed the contour map of the regression coefficients of four risk factors and their *p* values. It is clear that the regression coefficients varied spatially, and the local spatial relationship between iodine concentration and geographical factors exhibited a non-constant mean and variance across the whole area.

The regression coefficients of “Soil” were mostly negative (some were not statistically significant), except in the East area (Figure 5). The regression coefficients of “Distance to Yellow River” were negative in the east and north of Shandong Province, when the regression coefficients were positive in the other areas. The standardized regression coefficient estimates of “Hydrogeology” and “Topography” were not statistically significant (Figure 6).

## 4. Discussion

In this study, we mapped the distribution of iodine in drinking water at the county level in Shandong Province, China. Then spatial autocorrelation and spatial scan statistics analyses were conducted to investigate the highlighted geographic areas with significantly high concentrations of iodine. Besides, the GLMs and GWR were used to uncover the relationships between iodine concentration and geological factors. To our knowledge, this is the first study focusing on the spatial pattern of iodine in drinking water and relationships between iodine concentration and geological factors in Shandong Province.

The distribution of iodine in drinking water in Shandong Province was clustered, indicated by the significantly global Moran’s *I* (*I* = 0.52, *p* < 0.001). While, the Local Moran’s *I* and spatial scan statistic detected similar and significant high-risk clusterings. Both spatial cluster detection methods identified two nearly the same regions of high iodine, with one located in the south west (including nine counties) and the other located in the northwest (including nine by Local Moran’s *I* or 11 counties by spatial scan statistic). The two cluster areas were all located on the Yellow River flood plain. This finding should help policy makers to design better monitoring strategies, e.g., intensifying the surveillance in the cluster areas and draft suitable strategies for water improvement. It should be pointed out that only high-value clustering areas were detected in our study, although either iodine excess or deficiency can cause thyroid hormone disorders. Because the current iodine nutrition status of population was adequate and preliminary elimination of iodine-deficiency disorders was achieved since universal salt iodization (USI) implemented in China from 1995, including Shandong Province [33,34,35].

One possible reason for the high levels of iodine in drinking-water is that the Yellow River caused iodine deposits in the areas through flooding [36]. The iodine in drinking water mainly depended on the geological factors. In previous studies, researchers mainly focused on describing the geological characteristics in the high iodine areas, aiming at seeking the reasons for high iodine [37,38,39]. They found that the reasons for high iodine maybe related to the Yellow River. But in the present study, the dependence relationships between iodine concentration and geological factors were studied for the first time. GRW was used to analyze the local relationship between iodine concentration in drinking water and geological factors. The soil type and distance to Yellow River showed significant impacts on the iodine concentration. The iodine in drinking water in areas with cinnamon soil was higher than the other types. Generally, the iodine concentration decrease with the increasing of distance to the Yellow River. The distribution of cinnamon soil in Shandong Province was consistent with the high iodine areas. It was mainly located on the Yellow River flood plain, and it was caused by the Yellow River through flooding. This supported, to some extent, the hypothesis that Yellow River caused iodine deposits in the Shandong Province and their relationships showed obvious spatial variability reflected by the contour map of the regression coefficients of GWR model (Figure 5). To some extent, it was decided by the spatial distribution of the iodine in drinking water. Counties with iodine sufficiency were mostly located in west and east part, while counties with iodine deficiency were mostly located in the middle of Shandong Province. This indicated that the effect of geological factors on the concentration in drinking-water was different in different regions, suggesting that the local prevention strategies and monitoring schemes should be formulated according to the local association of the geological factors and iodine in drinking-water. Besides, the average R-square (0.63) of the GWR model was larger than the R-square (0.53) of GLM. It indicated that the fit of the GWR model was better than that of the GLM.

There are several limitations that deserve discussion. First, our spatial regression analysis was based on the county scale, but the distribution of iodine in drinking-water was also not homogeneous at the county level, so our study can’t identify the spatial heterogeneity within each unit. Secondly, the water iodine samples were investigated in two years, and were detected by 17 laboratories. So this might lead to some unexpected bias for the results, although standardized samples from the China External Quality Control were used. Thirdly, we did not assess the potential socioeconomic factors that could be associated with clustering since it is not a survey-based study.

## 5. Conclusions

The spatial distribution and cluster areas of iodine in drinking-water in Shandong Province were investigated and the relationship between the iodine concentration and geological factors were explored. The results showed that the distribution of iodine in drinking water in Shandong Province was clustered. Two high iodine cluster regions were detected, which suggested that more epidemiological and nutritional investigation should be conducted in the high iodine areas. Besides, the results indicated the “non-stationary” nature of the local relationship. Local prevention strategies and monitoring schemes should be formulated according to the local association of the geological factors and iodine in drinking-water. More important, our research showed that soil type and distance to the main course of the Yellow River were the main factors for the iodine concentration in drinking water. This can provide useful information to explore the reasons for the presence of high iodine levels in Shandong Province.
